# Increase in intra-abdominal pressure during airway suctioning-induced cough after a successful spontaneous breathing trial is associated with extubation outcome

**DOI:** 10.1186/s13613-018-0410-x

**Published:** 2018-05-08

**Authors:** Yasuhiro Norisue, Jun Kataoka, Yosuke Homma, Takaki Naito, Junpei Tsukuda, Kentaro Okamoto, Takeshi Kawaguchi, Lonny Ashworth, Shimada Yumiko, Yuiko Hoshina, Eiji Hiraoka, Shigeki Fujitani

**Affiliations:** 1 Department of Emergency and Critical Care Medicine, Tokyo Bay Urayasu Ichikawa Medical Center, 3-4-32 Todaijima, Urayasu, Chiba 2790001 Japan; 2 Department of Emergency and Critical Care Medicine, St. Marianna University Hospital, 2-16-1 Sugao, Kawasaki, Kanagawa 2168511 Japan; 3 Department of Respiratory Care, Boise State University, 1910 W University Drive, Boise, ID 83725 USA; 4 Department of Pulmonary and Critical Care Medicine, Tokyo Bay Urayasu Ichikawa Medical Center, 3-4-32 Todaijima, Urayasu, Chiba 2790001 Japan

**Keywords:** Cough, Airway suctioning, Extubation, Intra-abdominal pressure, Mechanical ventilation

## Abstract

**Background:**

A patient’s ability to clear secretions and protect the airway with an effective cough is an important part of the pre-extubation evaluation. An increase in intra-abdominal pressure (IAP) is important in generating the flow rate necessary for a cough. This study investigated whether an increase from baseline in IAP during a coughing episode induced by routine pre-extubation airway suctioning is associated with extubation outcome after a successful spontaneous breathing trial (SBT).

**Methods:**

Three hundred thirty-five (335) mechanically ventilated patients who passed an SBT were enrolled. Baseline IAP and peak IAP during successive suctioning-induced coughs were measured with a fluid column connected to a Foley catheter.

**Results:**

Extubation was unsuccessful in 24 patients (7.2%). Unsuccessful extubation was 3.40 times as likely for patients with a delta IAP (ΔIAP) of ≤ 30 cm H_2_O than for those with a ΔIAP > 30 cm H_2_O, after adjusting for APACHE II score (95% CI, 1.39–8.26; *p* = .007).

**Conclusion:**

ΔIAP during a coughing episode induced by routine pre-extubation airway suctioning is significantly associated with extubation outcome in patients with a successful SBT.

*Trial registration* UMIN-CTR Clinical Trial, UMIN000017762. Registered 1 June 2015.

## Background

Although cough strength for clearing secretions is important in successful extubation, it is not routinely objectively evaluated in daily practice after a successful spontaneous breathing trial (SBT). The inability to produce an adequate cough—because of muscle weakness or pain—increases the risks of atelectasis, oxygen desaturation, re-intubation, and, possibly, pneumonia [1–3].

Cough strength, as measured by voluntary and involuntary cough peak expiratory flow (CPEF), has been proposed as an independent predictor of successful extubation [4–10]. Previous studies reported that an involuntary CPEF of < 60 L/min was significantly associated with increased risk of extubation failure [5, 9, 10]. In addition to methods that focus on CPEF, clinicians desire a procedure that would allow evaluation of involuntary cough strength among patients who are unable or unwilling to produce maximal cough effort without special devices. Ideally, this procedure would not require disconnecting the patient from the ventilator circuit during routine pre-extubation airway suctioning, as this almost always induces cough.

Physiologically, a cough begins with a deep inspiratory phase, followed by an expiratory phase of bursts of intercostal and abdominal muscle contractions [11]. This results in “the compressive phase” and an abrupt rise in intrapleural and intra-abdominal pressure (IAP) [12] with the relaxed diaphragm. IAP is then transmitted into intrapleural pressure [13], which abruptly increases airway pressure and cough. The increase in intra-abdominal pressure (ΔIAP) during an episode of continuous coughing is thus positively correlated with cough strength [13–16]. Use of a Foley catheter to measure bladder pressure during cough is straightforward and can be performed in most centers. We tested the hypothesis that low ΔIAP is associated with extubation failure after a successful SBT.

## Methods

### Study design

This is a single-center, prospective, cohort study. The study was approved by the Institutional Review Board at Tokyo Bay Urayasu Ichikawa Medical Center (TBUIMC). A waiver of informed consent was obtained because the study exposed patients to less than minimal risk.

### Patients

The study was performed in the medical–surgical ICU during the period from April 2015 through November 2015. All mechanically ventilated patients 18 years or older who had been endotracheally intubated and had passed an SBT of longer than 30 min were eligible for inclusion. The SBT was conducted on pressure support ventilation with a pressure support of 5 cm H_2_O, a positive end-expiratory pressure (PEEP) of ≤ 8 cm H_2_O, and a fraction of inspiratory oxygen (FiO_2_) of ≤ 0.50. Patients were excluded from the study if they had “comfort care” or “do not re-intubate” status or had been previously extubated during the same hospitalization. Patients were also excluded if they had documented or suspected upper airway obstruction, end-stage renal disease requiring hemodialysis, or no Foley catheter at the time of extubation. Successful completion of an SBT was determined using the standard Tokyo Bay Urayasu Ichikawa Medical Center (TBUIMC) Respiratory Care Weaning Protocols (no evidence of severe anxiety, dyspnea, or excessive accessory muscle use; a rapid shallow breathing index [RSBI] of ≤ 105 breaths/min/L; and adequate gas exchange, i.e., SaO_2_ ≥ 90% with FiO_2_ ≤ 0.50 and PEEP ≤ 8 cm H_2_O).

### Observations and measurements

A water-column technique was used to measure IAP [17], which was determined in the ICU by resident physicians using the following protocol, after all sedatives and analgesics were discontinued for at least 60 min: (1) the drainage tube of the patient’s Foley bladder catheter was clamped; (2) sterile normal saline (20 ml) was instilled into the bladder via the aspiration port of the Foley catheter with a needleless connection system; (3) a fluid column consisting of two extension tubes (length 75 cm, inner diameter 3.1 mm; Terumo, Tokyo, Japan) was constructed, connected to the aspiration port of the Foley catheter, and then placed at the level of the mid-axillary line; (4) with the patient in supine position, fluid level in the absence of cough at end expiration was marked on the extension tube and recorded as the baseline bladder pressure; (5) airway suctioning was performed by advancing the closed-system suction catheter while the patient was connected to the ventilator, which is part of the standard pre-extubation procedure; and (6) the recorder observed changes in fluid level and marked the highest fluid level on the extension tube during successive coughs, which was recorded as the highest bladder pressure. The patient was extubated within 10 min after IAP measurement. Attending physicians and fellows responsible for clinical decisions, including extubation, were blinded to the results of the IAP and ΔIAP measurements.

### Definitions of extubation success and failure

Successful extubation was defined as the absence of the need for re-intubation within 72 h after extubation. Extubation failure was defined as re-intubation within 72 h after extubation. Patients were followed until hospital discharge or death. The use of prophylactic or therapeutic noninvasive positive pressure ventilation without consequent re-intubation was not considered as extubation failure.

### Sample size

Because at least 10 episodes of extubation failure were required in order to conduct multiple regression analysis adjusted for APACHE II score—the most important confounding factor for extubation outcomes—the estimated minimum sample size needed for the statistical analysis was 135 with a predicted extubation failure rate of 8%, as indicated by the past extubation failure rate in this ICU [18]. With a planned study duration of 9 months, the predicted number of patients to be recruited in the study was 400, assuming an average of approximately 45 extubations per month in our ICU.

### Statistical analysis

The primary outcome of this study was extubation failure. Secondary outcomes included in-hospital mortality, ICU days, and length of hospital stay. A ΔIAP cutoff value for extubation failure was estimated with receiver operator characteristic (ROC) analysis. A multivariable-adjusted logistic regression model was used to calculate the odds ratio for extubation failure based on ΔIAP adjusted for APACHE II score. Mean baseline IAP, ΔIAP, and other variables were compared in relation to extubation success and failure. The Student *t* test was used to compare the means for variables. The Fisher exact test was used to compare grouped data such as sex, Confusion Assessment Method for the Intensive Care Unit (CAM-ICU), and mortality. For measures of association, 95% confidence intervals (CI) were computed, and statistical significance was defined as a two-tailed *p* value of less than .05. Using a multivariable-adjusted logistic regression model, we estimated the odds ratio (OR) for re-intubation adjusted for APACHE II score. We also conducted a secondary analysis to investigate the relationship between ΔIAP and extubation outcomes in patients who were mechanically ventilated for longer than 72 h. All statistical analyses, except for sample size estimation, were performed with the IBM Statistical Package for the Social Sciences version 22.0 (IBM, Corp, Armonk, NY, USA).

## Results

### Patients

**Fig. 1:**
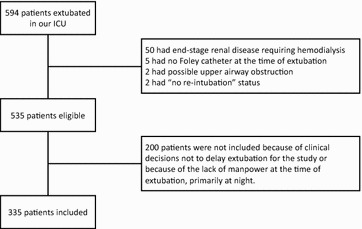
Flowchart of the study

**Fig. 2:**
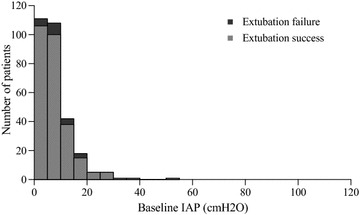
Histogram showing the number of patients and baseline intra-abdominal pressure (IAP)

**Fig. 3:**
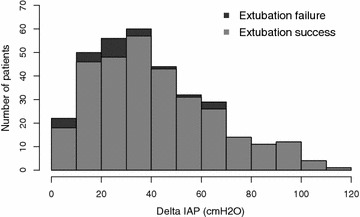
Histogram showing the number of patients and Δintra-abdominal pressure (ΔIAP)

A total of 335 patients were included in the analyses (Fig. 1), 24 (7.2%) of whom were re-intubated within 72 h after extubation. Tables 1 and 2 show patient baseline characteristics and indications for intubation, respectively. Univariate analysis showed that CAM-ICU, APACHE II score, Simplified Acute Physiology Score II score, intubation days, length of ICU stay, length of hospital stay, 28-day mortality, and in-hospital mortality were significantly higher, and P/F ratio was significantly lower, in the extubation failure group than in the extubation success group. Figures 2 and 3 show the distributions of baseline IAP and ΔIAP for the patients. The median (interquartile range) baseline IAP was 8 (4–11) cm H_2_O, and the median (interquartile range) ΔIAP was 38 (23–55) cm H_2_O (range, 0–120 cm H_2_O).

**Table 1:** Baseline characteristics of patients in each group

Characteristics	Extubation success	Extubation failure	*p* value
Number of patients,* n*	311	24	
Male sex,* n* (%)	193 (62.1)	17 (70.8)	0.512
Age, median (IQR)	71 (62–79)	72 (64–78)	0.581
BMI, median (IQR)	22.7 (20.3–25.2)	21.05 (17.3–24.8)	0.115
GCS, median (IQR)	11 (10–11)	11 (10–11)	0.667
CAM-ICU, positive (%)	40 (12.9)	8 (33.3)	0.012
APACHE II score, median (IQR)	20 (17–24)	24 (22–28)	< 0.001
SAPS II score, median (IQR)	41 (32–51)	51 (46–59)	< 0.001
In–out balance, median ml (IQR)	2959 (1000–5322)	2676 (793–4500)	0.701
Intubation days, median (IQR)	2 (1–3)	4 (2–6)	0.001
P/F ratio, median (IQR)	300 (250–367)	275 (218–326)	0.036
TV, median L (IQR)	0.44 (0.36–0.55)	0.47 (0.40–0.66)	0.139
MV, median L (IQR)	6.90 (5.60–8.19)	7.90 (5.76–9.75)	0.087
RSBI, median breaths/min/L (IQR)	37.2 (26.4-48.9)	38.2(16.9-51.3)	0.691
Length of ICU stay, median (IQR)	4 (2–6)	12 (6–16)	< 0.001
Length of hospital stay, median (IQR)	20 (14–36)	48 (27–55)	< 0.001
28-Day mortality,* n* (%)	3 (1.0)	3 (12.5)	0.006
In-hospital mortality,* n* (%)	10 (3.2)	5 (20.8)	0.002
Baseline IAP, mm H_2_0, median (IQR)	7.9 (4.0–10.0)	8.0 (5.7–13.0)	0.19
ΔIAP, mm H_2_O, median (IQR)	39.0 (24.0–57.0)	25.5 (19.8–38.3)	0.012

**Table 2:** Indications for intubation in each group

Indications for intubation	Extubation success (*n*)	Extubation failure (*n*)	*p* value
Emergent abdominal surgery	13	1	0.22
Emergent non-abdominal surgery	48	5	
Elective abdominal surgery	10	1	
Elective non-abdominal surgery	110	3	
Altered mental status	3	0	
Acute myocardial infarction	7	1	
Congestive heart failure	19	0	
Asthma	1	0	
Pneumonia	13	1	
Sepsis	22	3	
COPD	2	1	
Drug intoxication	5	0	
Hemorrhagic stroke	9	1	
Ischemic stroke	2	0	
Gastrointestinal bleeding	4	0	
Status epilepticus	6	1	
Others	37	6

### ΔIAP and outcome measures

**Fig. 4:**
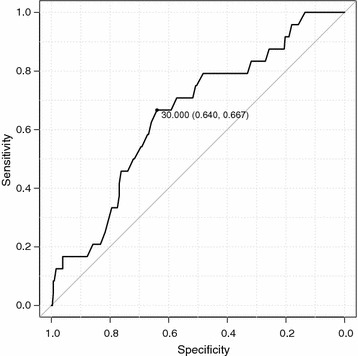
ROC curve between extubation failure and Δintra-abdominal pressure (ΔIAP)

ΔIAP was significantly higher in the extubation success group than in the extubation failure group (*p* = 0.012; median, 39.00 vs 25.50 cm H_2_O, respectively). Figure 4 shows the ROC curve between ΔIAP and extubation failure. The area under the ROC curve was 0.654 (95% CI 0.544–0.764), and the cutoff value was 30 cm H_2_O (sensitivity, 64%; specificity, 67%). ΔIAP was classified as ≤ 30 cm H_2_O (low ΔIAP group) or > 30 cm H_2_O (high ΔIAP group). Table 3 shows that low ΔIAP was significantly associated with extubation failure after adjusting for APACHE II score (adjusted OR, 3.40; 95% CI, 1.39–8.26, *p* = .007). The positive predictive value and negative predictive value of a ΔIAP value of ≤ 30 cm H_2_O for extubation failure were 1.85 and 0.52, respectively.

**Table 3:** Unadjusted and adjusted odds ratio of low ΔIAP for extubation failure

	OR	95% CI	*p* value
Unadjusted	3.56	1.47–8.55	0.005
Adjusted*	3.40	1.39–8.26	0.007

*Adjusted for APACHE II score

### ΔIAP and outcome measures in patients who were mechanically ventilated for longer than 72 h

**Fig. 5:**
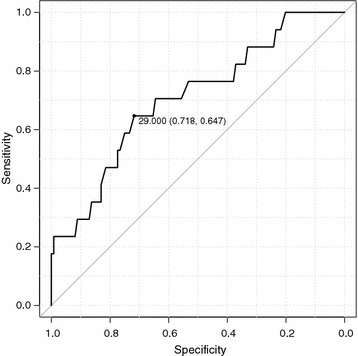
ROC curve between extubation failure and Δintra-abdominal pressure (ΔIAP) in patients who were mechanically ventilated for more than 72 h

A secondary analysis including only patients who were mechanically ventilated for longer than 72 h (124 patients with successful extubation and 17 patients with extubation failure) yielded an AUC of 0.708 (95% CI 0.571–0.845) with a cutoff value of 29 cm H_2_O (Fig. 5). Multiple regression analysis (Table 4) showed that a low ΔIAP (≤ 29 cm H_2_O) was significantly associated with extubation failure, after adjusting for APACHE II score (adjusted OR, 3.79; 95% CI, 1.32–10.75, *p* = 0.01). The positive predictive value and negative predictive value of a ΔIAP value of ≤ 29 cm H_2_O for extubation failure were 2.21 and 0.56, respectively.

**Table 4:** Unadjusted and adjusted odds ratio of low ΔIAP for extubation failure in patients were mechanically ventilated for more than 72 h

	OR	95% CI	*p* value
Unadjusted	3.93	1.39–11.20	0.01
Adjusted*	3.79	1.32–10.75	0.01

*Adjusted for APACHE II score

## Discussion

This study showed that diminished ΔIAP during coughing induced by routine pre-extubation suctioning was significantly associated with extubation failure. Expiratory muscle strength is important in producing a successful cough [19]. However, CPEF is the only widely accepted method of evaluating pre-extubation expiratory muscle strength during cough production. Smina et al. [5] reported that a CPEF ≤ 60 L/min yielded an AUC of 0.7 (sensitivity 69%; specificity 74%) in predicting extubation failure. Our ΔIAP data indicated similar predictive values, especially in patients mechanically ventilated for longer than 72 h. These findings suggest that ΔIAP is a potentially useful parameter for assessing expiratory muscle strength.

The cough reflex protects the airway by means of a continuous series of expiratory coughs with subsequent inspiratory efforts [20–22]. The continuous increase in IAP during such an episode provides sustained expiratory force [14]. The present results are consistent with these physiological characteristics of the cough reflex and support the hypothesis that an inability to increase IAP predicts extubation failure. Moreover, our secondary analysis of patients intubated for longer than 72 h yielded a better AUC in predicting extubation failure. The present results are attributable to the significant association between duration of mechanical ventilation and ICU-acquired weakness (ICUAW) [23]; thus, our method might be more relevant and useful for patients at high risk of ICUAW, including expiratory muscle weakness.

The proposed method of estimating cough strength has several practical strengths. Most mechanically ventilated patients already have a Foley catheter, and IAP measurement is feasible in most ICUs. Second, airway suctioning is part of the pre-extubation process; therefore, ΔIAP measurement can be included in routine pre-extubation evaluation. Finally, cough strength induced by airway suctioning does not depend on patient effort and is thus feasible for most mechanically ventilated patients, including those who are uncooperative because of dementia, delirium, or altered mental status.

Future studies should investigate how to apply ΔIAP to clinical decision making. For example, a patient who has passed an SBT but has a low ΔIAP may need appropriate preparation for possible re-intubation. Unlike the present patients with a low ΔIAP, none of those with a ΔIAP > 70 cm H_2_O had extubation failure (Fig. 3). Thus, a ΔIAP > 70 cm H_2_O may be potentially used to exclude the possibility of extubation failure in patients with a successful SBT and no airway obstruction.

This study has limitations that warrant mention. ΔIAP was measured with a fluid column rather than by connecting the Foley catheter to a digital pressure transducer. Because of resistance in the extension tube, the fluid level might not have reached the true maximum pressure level during a coughing episode. Moreover, the accuracy of visual IAP measurement has not been validated and may not be accurate. The present cutoff value might therefore be more accurately regarded as a cutoff value for the fluid column method than as the true ΔIAP cutoff value.

## Conclusion

In conclusion, ΔIAP during a coughing episode induced by routine pre-extubation airway suctioning is significantly associated with extubation outcome in patients with a successful SBT.

## Abbreviations

APACHE: Acute Physiology and Chronic Health Evaluation
CAM-ICU: Confusion Assessment Method for the Intensive Care Unit
CI: confidence interval
ESRD: end-stage renal disease
FiO2: fraction of inspired oxygen
GCS: Glasgow Coma Scale
IAP: intra-abdominal pressure
ICU: intensive care unit
OR: odds ratio
PEEP: positive end-expiratory pressure
PEF: peak expiratory flow
ROC: receiver operator characteristic
RSBI: rapid shallow breathing index
SBT: spontaneous breathing trial
SAPS: Simplified Acute Physiology Score
TBUIMC: Tokyo Bay Urayasu Ichikawa Medical Center
